# RFC2: a prognosis biomarker correlated with the immune signature in diffuse lower-grade gliomas

**DOI:** 10.1038/s41598-022-06197-5

**Published:** 2022-02-24

**Authors:** Xu Zhao, Yuzhu Wang, Jing Li, Fengyi Qu, Xing Fu, Siqi Liu, Xuan Wang, Yuchen Xie, Xiaozhi Zhang

**Affiliations:** Department of Radiation Oncology, The First Affiliated Hospital of Xi’an Jiaotong University, No.277, Yanta West Road, Xi’an, 710061 Shaanxi China

**Keywords:** Cancer, Cancer microenvironment, CNS cancer, Tumour immunology, Prognostic markers

## Abstract

Diffuse lower-grade gliomas (LGG) represent the highly heterogeneous and infiltrative neoplasms in the central nervous system (CNS). Replication factor C 2 (RFC2) is a subunit of the RFC complex that modulates DNA replication and repair. However, the prognosis value of RFC2 and its association with the immune signature of tumor microenvironment (TME) in LGG remains unknown. Based on Oncomine, TCGA, GTEx, TIMER, GEPIA, and HPA databases, we evaluated RFC2 expression levels and its clinical prognostic value in LGG and other cancers. Then we analyzed the correlations between RFC2 expression and tumor mutation burden (TMB), tumor microsatellite instability (MSI), and mismatch repair (MMR) genes across cancers. And CIBERSORT and ESTIMATE algorithms were conducted to estimate the association of RFC2 with immune cell infiltration of LGG. Additionally, we performed the functional enrichment analyses of RFC2 in LGG. Then functional experiments were employed to further validate the oncogenic role of RFC2 in LGG. Our results showed that RFC2 was widely highly expressed in most types of cancer. And its expression was closely related to the clinicopathological features and prognosis in LGG and other cancer types. RFC2 levels were also correlated with TMB and MSI across various cancers. Furthermore, RFC2 was positively associated with the infiltration levels of immune cells and immune checkpoint genes in LGG. Additionally, in vitro experiments revealed that RFC2 played an oncogenic role in LGG progression. In conclusion, our findings revealed that RFC2 could serve as a reliable biomarker to predict the prognosis and immune signature for LGG.

## Introduction

Gliomas are among the most frequent fatal malignant tumors in the central nervous system (CNS), which account for approximately 70% of all primary brain and CNS tumors^1^. According to molecular genetics and histopathological features, the World Health Organization (WHO) mainly classified gliomas into diffuse lower-grade gliomas (LGG) and glioblastoma (GBM) multiforme^2^. LGG comprises diffuse low-grade (WHO grade II) and intermediate-grade (WHO grade III) gliomas, including astrocytomas, oligodendrogliomas, and mixed oligoastrocytomas base on pathological types^3^. Although LGG shows some sensitivity to current standard therapy, such as surgery combined with radiotherapy and chemotherapy, its prognosis remains frustrating due to its inevitable progression and treatments resistance^4,5^. Thus, it is of great profound to identify a potential prognostic and therapeutic target for improving the outcomes of LGG patients.

In recent years, cancer immunotherapy, a novel strategy that aims to activate and boost the immune system to directly recognize and eliminate tumor cells, has achieved tremendous development, which has been regarded as a promising practice for cancer treatment^6,7^. More recently, the immune checkpoint blockade (ICB) therapies, including cytotoxic T lymphocyte antigen-4 (CTLA-4), programmed death-1 (PD-1) and programmed death ligand-1 (PD-L1) inhibitors, as well as chimeric antigen receptor (CAR) T cells have been widely approved for clinical use in many types of cancer, such as melanoma, non-small cell lung cancer (NSCLC), classical Hodgkin Lymphoma, and urothelial bladder cancer, and revealed a robust anti-tumor effect in these malignancies^8,9^. However, these representative immune therapies show less favorable efficacy in LGG, as it hijacks immune checkpoints to escape immune surveillance through its harsh tumor microenvironment (TME)^10^. Increasing evidence has indicated that the immunosuppressive environment mediated by tumor-infiltrating immune cells (TICs), such as regulatory T (Treg) cells and tumor-associated macrophages (TAMs), hinders the delivery of immunotherapies in LGG^11^. In this context, a better understanding of the unique immune signature of TME and exploration of immune-related biomarkers in LGG are urgently required.

Replication factor C (RFC) is a structure-specific protein complex consisting of 5 subunits (RFC1-5), which functions as a primer recognition factor for DNA polymerase to regulate DNA replication and repair through binding with DNA^12^. Increasing evidence reveals that the RFC complex plays a vital role in cancer progression and therapeutic resistance. For example, RFC1 interacts with proliferating cell nuclear antigen (PCNA) to promote breast cancer cell survival^13^. Overexpression of RFC3 results in an increased invasion and migration in lung adenocarcinoma cells^14^. RFC4 enhances DNA damage repair mediated by non-homologous end joining to protect colorectal cancer cells from ionizing radiation-induced apoptosis^15^. Moreover, the upregulation of RFC5 transcriptionally activated by Forkhead box M1 (FOXM1) leads to temozolomide resistance in glioma cells^16^. Among them, the RFC2 gene is located within human chromosome 7q11.23, which encodes a 40 kDa subunit in the RFC complex^17^. Recently, some reports demonstrated that the aberrant expression of RFC2 was closely involved in the progression and metastasis of several cancers. For instance, RFC2 expression was elevated in colorectal cancer (CRC) tissues and related to aggressive CRC clinicopathological symptoms, and its knockdown significantly suppressed the proliferation of CRC cells^18^. In addition, the abnormal overexpression of RFC2 was found to promote the invasion and migration in hepatocellular carcinoma (HCC)^19^. Nevertheless, the functional roles of RFC2 in the regulation of progression and TME in LGG have not been fully elucidated.

Therefore, in this study, we aimed to investigate RFC2 expression levels and their associations with the clinicopathological parameters and prognosis in LGG and other different types of cancer by using various databases, including Oncomine, The Cancer Genome Atlas (TCGA), Genotype-tissue expression (GTEx), Tumor Immune Estimation Resource (TIMER), Gene Expression Profiling Interactive Analysis (GEPIA), and Human Protein Atlas (HPA). Further, we also analyzed the correlations between RFC2 expression and tumor mutation burden (TMB), tumor microsatellite instability (MSI), and mismatch repair (MMR) genes across pan-cancer. Moreover, we comprehensively examined the potential relationship between RFC2 and the immune phenotype of TME in LGG by conducting CIBERSORT and ESTIMATE algorithm. Additionally, we performed Gene Ontology (GO), Kyoto Encyclopedia of Genes and Genomes (KEGG), Reactome enrichment analyses, as well as Gene Set Enrichment Analysis (GSEA) to explore the functional role of RFC2 in LGG. Moreover, in vitro experiments were conducted to verify the potential effects of RFC2 on LGG progression. In general, our results suggested that RFC2 served as an effective prognostic marker in LGG, and provided a potential therapeutic target for LGG immunotherapy.

## Results

### RFC2 expression analysis in pan-cancer

**Figure 1:**
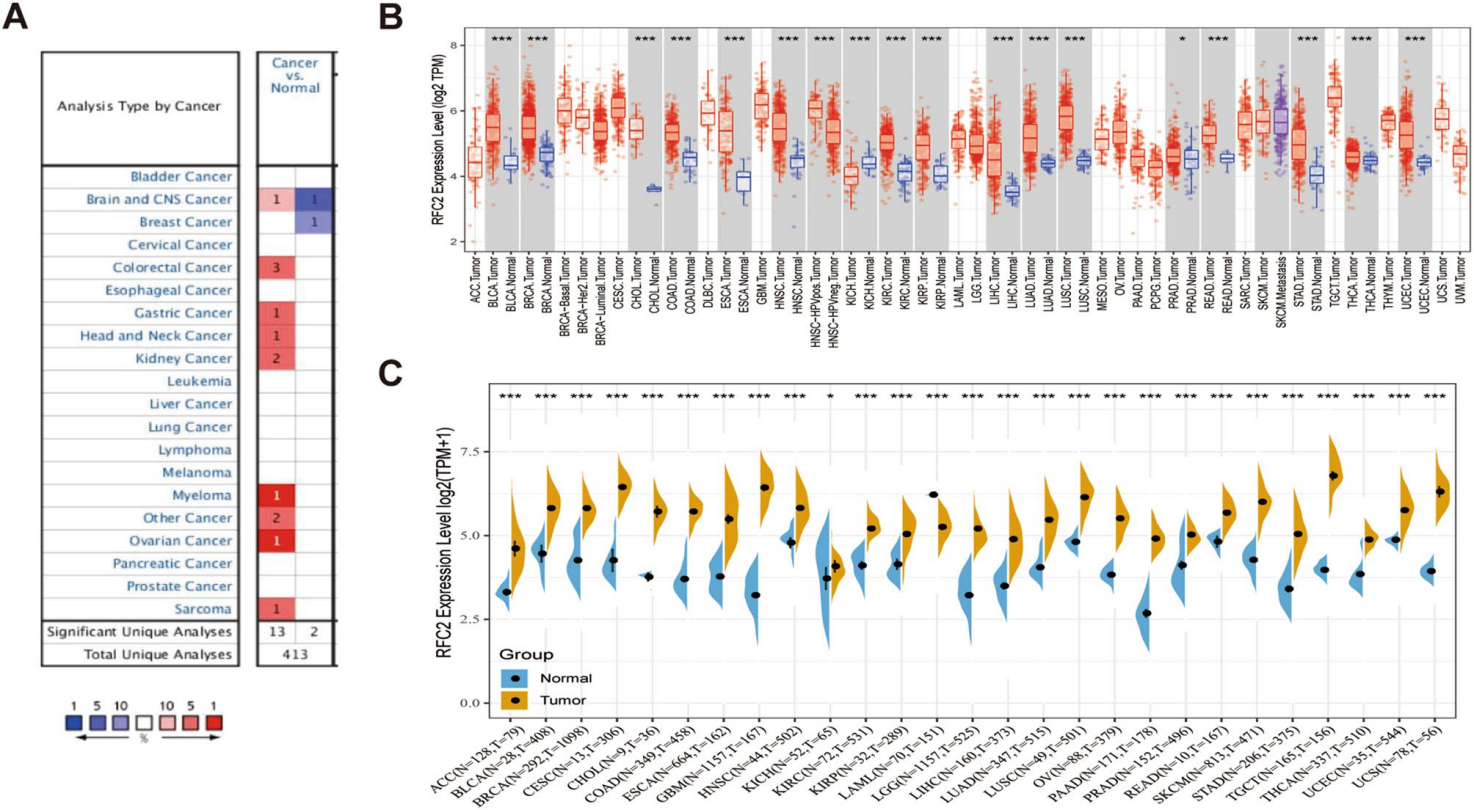
RFC2 expression levels in pan-cancer. (**A**) RFC2 expression in cancer tissues compared to normal tissues in Oncomine database. For each pair, red indicated higher expression and blue indicated lower expression. (**B**) The expression levels of RFC2 in different cancer types from TCGA database were analyzed by TIMER database. (**C**) The differential expression of RFC2 in tumor tissues from TCGA database was compared with normal tissues from GTEx database. (*P < 0.05, **P < 0.01, ***P < 0.001).

Firstly, we examined RFC2 expression levels in pan-cancer tissues based on Oncomine analysis. The results showed that RFC2 was generally highly expressed in many types of cancer tissues compared with normal tissues, including brain and CNS cancer, colorectal cancer, gastric cancer, head and neck cancer, kidney cancer, myeloma, ovarian cancer, sarcoma, and other cancer (Fig. 1A). Then we evaluated the differential expression of RFC2 in TCGA dataset by using TIMER online database. As shown in Fig. 1B, the significant higher expression levels of RFC2 were observed in bladder urothelial carcinoma (BLCA), breast invasive carcinoma (BRCA), cholangiocarcinoma (CHOL), colon cancer (COAD), esophageal carcinoma (ESCA), head and neck squamous cell carcinoma (HNSC), kidney chromophobe (KICH), kidney renal clear cell carcinoma (KIRC), kidney renal papillary cell carcinoma (KIRP), liver cancer (LIHC), lung adenocarcinoma (LUAD), lung squamous cell carcinoma (LUSC), prostate adenocarcinoma (PRAD), rectum adenocarcinoma (READ), stomach adenocarcinoma (STAD), thyroid carcinoma (THCA), and uterine corpus endometrial carcinoma (UCEC). Given the limitations of normal samples in TCGA database, we matched the normal tissues of Genotype-Tissue Expression (GTEx) with the cancer tissues of TCGA to fully reflect the expression landscape of RFC2. The results in Fig. 1C showed that the expression levels of RFC2 gene were significantly increased in adrenocortical carcinoma (ACC), BLCA, BRCA, cervical cancer (CESC), CHOL, COAD, ESCA, GBM, HNSC, KICH, KIRC, KIRP, LGG, LIHC, LUAD, LUSC, ovarian cancer (OV), pancreatic cancer (PAAD), PRAD, READ, skin cutaneous melanoma (SKCM), STAD, testicular germ cell tumor (TGCT), THCA, UCEC, and uterine carcinosarcoma (UCS). In contrast, the RFC2 gene had a much lower expression in acute myeloid leukemia (LAML) (Fig. 1C).

**Figure 2:**
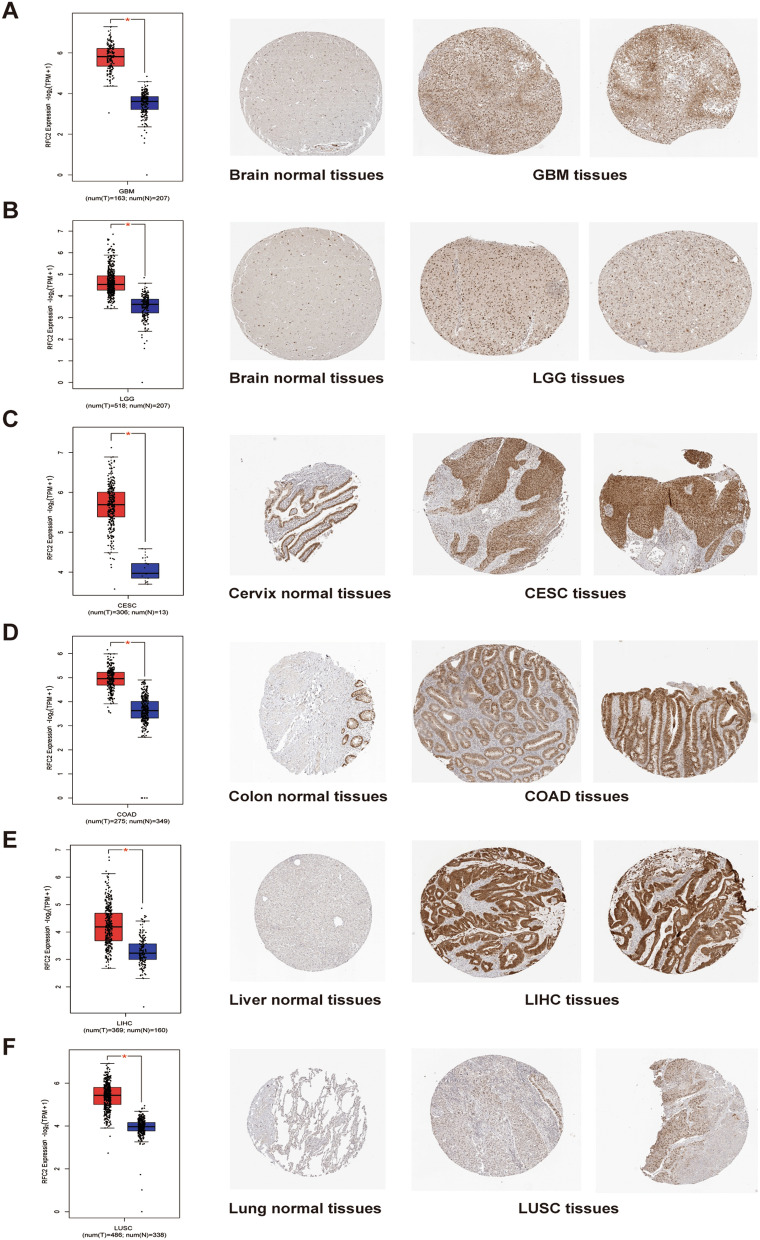
Comparison of RFC2 expression between tumor and normal tissues (left, red represented tumor tissues while blue represented normal tissues) and corresponding immunohistochemistry images in normal tissues (middle) and tumor tissues (right). (**A**–**F**) The differential expression levels of RFC2 in glioblastoma (GBM) tissues (**A**), diffuse lower-grade gliomas (LGG) tissues (**B**), cervical cancer (CESC) tissues (**C**), colon adenocarcinoma (COAD) tissues (**D**), liver hepatocellular carcinoma (LIHC) tissues (**E**), lung squamous cell carcinoma (LUSC) tissues (**F**), and their corresponding normal tissues were evaluated based on GEPIA and HPA databases. (*P < 0.05).

Further, RFC2 protein expression levels were evaluated based on immunohistochemical staining results provided by HPA and GEPIA databases. The results revealed a significant differential expression of RFC2 protein between multiple cancer tissues and corresponding normal tissues. Specifically, the RFC2 protein levels were obviously up-regulated in GBM tissues, LGG tissues, CESC tissues, COAD tissues, LIHC tissues, and LUSC tissues compared with their corresponding normal tissues (Fig. 2A–F). These results suggested that RFC2 was a potential biomarker for the malignancy of multiple cancers, including LGG.

### The correlation between RFC2 expression and clinicopathological characteristics in primary LGG patients

**Figure 3:**
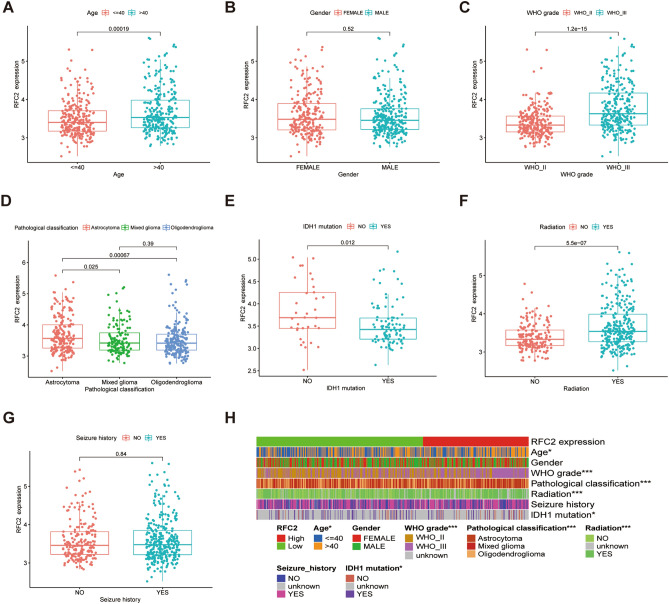
The correlation between RFC2 expression and clinicopathological characteristics in primary LGG patients (TCGA cohort, n = 537). (**A**–**G**) The correlations of RFC2 expression with LGG clinicopathological features, including age (**A**), gender (**B**), WHO grade (**C**), pathological classification (**D**), IDH1 mutation status (**E**), radiation therapy history (**F**), and seizure history (**G**). (**H**) Heatmap showing the association between RFC2 expression and clinicopathological characteristics in LGG patients. (*P < 0.05, **P < 0.01, ***P < 0.001).

As the aberrant high expression of RFC2 in LGG, we next analyzed the correlation between RFC2 expression and clinicopathological characteristics in primary LGG patients from TCGA dataset. The results showed that RFC2 expression in the > 40 years age group was significantly higher than that in the ≤ 40 years age group (*P* = 0.00019) (Fig. 3A). While there was no apparent association between RFC2 levels and LGG patients’ gender (*P* = 0.52) (Fig. 3B). Based on WHO grade and pathological classification, a distinctively elevated expression of RFC2 was observed in LGG patients with WHO III grade (*P* < 0.001) and astrocytoma (*P* = 0.025 and *P* = 0.00067 respectively) (Fig. 3C,D). Besides, RFC2 was highly expressed in the IDH1 wild-type group than that in the IDH1 mutation group (*P* = 0.012) (Fig. 3E). In addition, the expression levels of RFC2 were remarkably increased in LGG patients receiving radiation therapy (*P* < 0.001) (Fig. 3F). However, no significant correlation between RFC2 expression and seizure history of LGG patients was observed (*P* = 0.84) (Fig. 3G). Taken together, the differential expression of RFC2 was significantly correlated with age, WHO grade, pathological classification, IDH1 mutation status, and radiation therapy of LGG patients, while no obvious association with gender and seizure history (Fig. 3H). These results indicated that the abnormal expression of RFC2 was correlated with the malignant clinicopathological features of LGG.

### The prognostic value of RFC2 across LGG and other cancers

**Figure 4:**
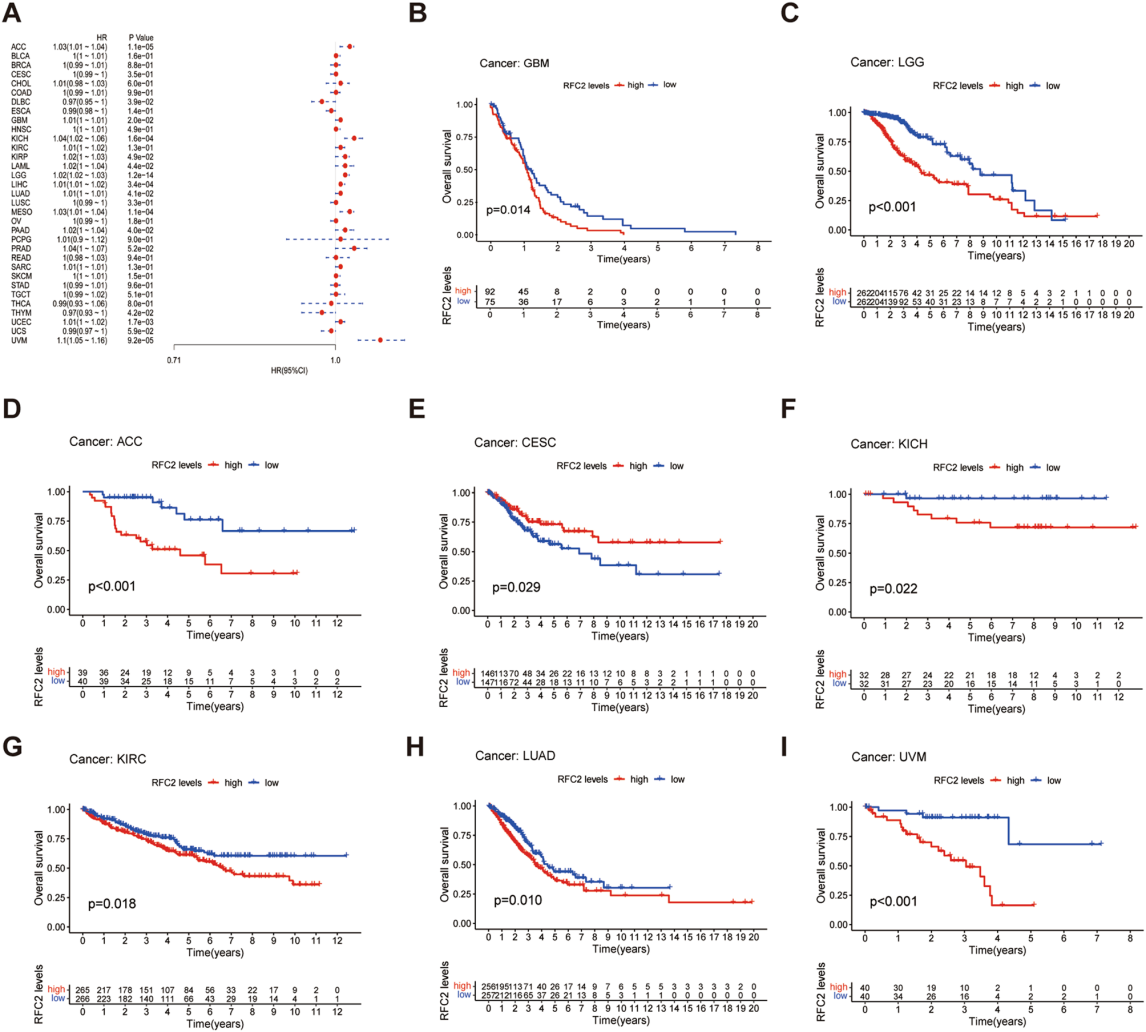
Association between RFC2 expression and overall survival (OS) across cancers in TCGA cohort. (**A**) Forest plot showing the correlation of RFC2 expression with OS in 33 types of cancer. (**B**–**I**) Kaplan–Meier survival analyses displaying the relationships of RFC2 expression with OS in different cancer types, including glioblastoma (GBM) (**B**), diffuse lower-grade gliomas (LGG) (**C**), adrenocortical carcinoma (ACC) (**D**), cervical cancer (CESC) (**E**), kidney chromophobe (KICH) (**F**), kidney renal clear cell carcinoma (KIRC) (**G**), lung adenocarcinoma (LUAD) (**H**), and ocular melanomas (UVM) (**I**).

To further explore the potential value of RFC2 in predicting the prognosis of LGG and other cancers, we analyzed the associations between RFC2 expression and overall survival (OS), disease-specific survival (DSS), disease-free interval (DFI), and progression-free interval (PFI) across cancers in TCGA cohort. The *Cox* proportional hazards model analysis revealed that the expression levels of RFC2 were correlated with the OS in ACC, lymphoid neoplasm diffuse large B-cell lymphoma (DLBC), GBM, KICH, KIRP, LAML, LGG, LIHC, LUAD, mesothelioma (MESO), PAAD, thymoma (THYM), UCEC, and ocular melanomas (UVM) (Fig. 4A). Besides, RFC2 served as a high-risk gene in ACC, GBM, KICH, KIRP, LAML, LGG, LIHC, LUAD, MESO, PAAD, UCEC, and UVM, while it acted as a low-risk gene in DLBC and THYM. Furthermore, Kaplan–Meier survival analysis also showed that patients with high levels of RFC2 in GBM (*P* = 0.014) (Fig. 4B), LGG (*P* < 0.001) (Fig. 4C), ACC (*P* < 0.001) (Fig. 4D), KICH (*P* = 0.022) (Fig. 4F), KIRC (*P* = 0.018) (Fig. 4G), LUAD (*P* = 0.01) (Fig. 4H), and UVM (*P* < 0.001) (Fig. 4I) had an apparently worse OS. However, higher expression of RFC2 was significantly associated with an increased OS in CESC (*P* = 0.029) (Fig. 4E).

**Figure 5:**
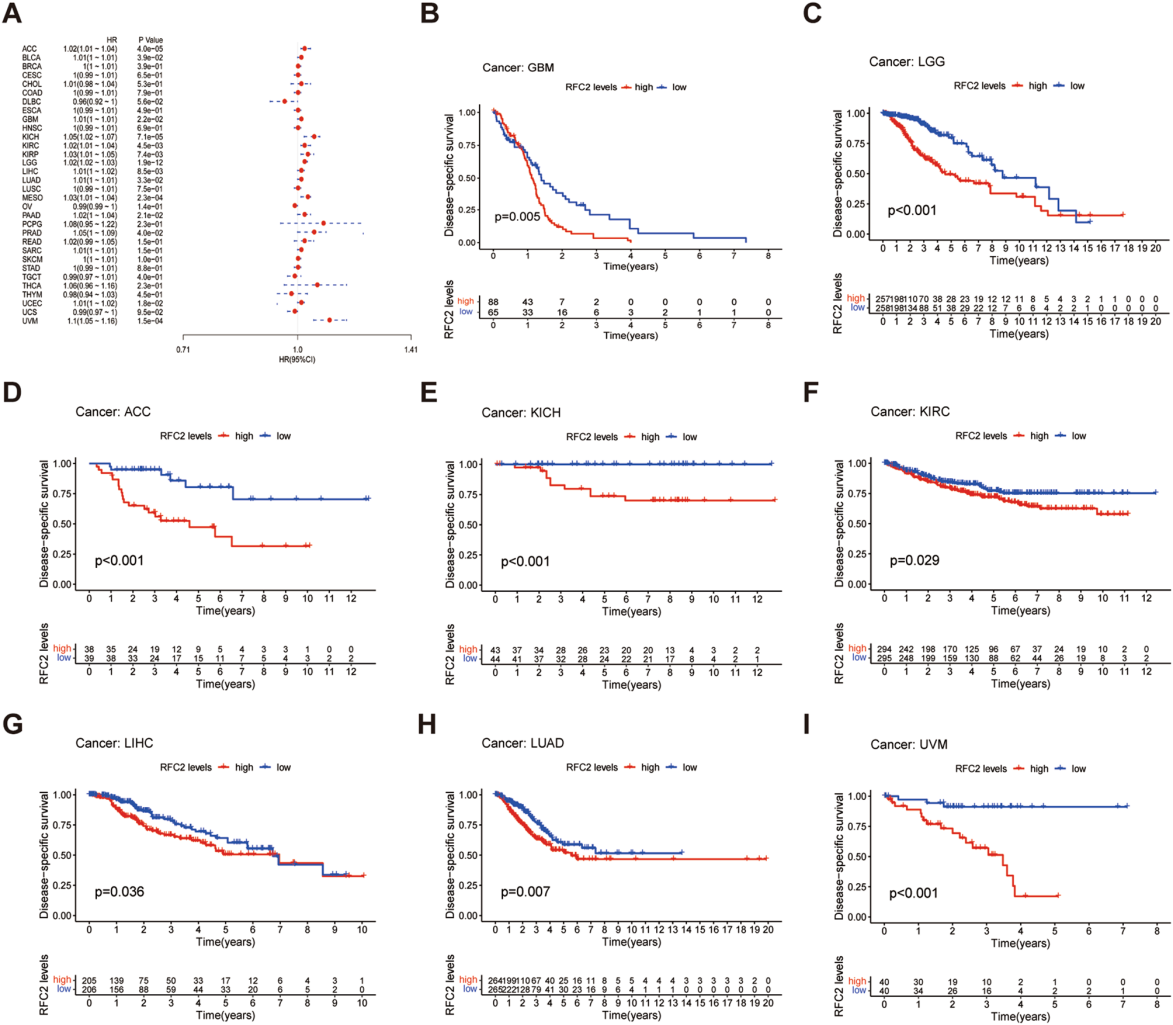
Association between RFC2 expression and disease-specific survival (DSS) across cancers in TCGA cohort. (**A**) Forest plot showing the correlation of RFC2 expression with DSS in 33 types of cancer. (**B**–**I**) Kaplan–Meier survival analyses displaying the relationships of RFC2 expression with DSS in different cancer types, including glioblastoma (GBM) (**B**), diffuse lower-grade gliomas (LGG) (**C**), adrenocortical carcinoma (ACC) (**D**), kidney chromophobe (KICH) (**E**), kidney renal clear cell carcinoma (KIRC) (**F**), liver hepatocellular carcinoma (LIHC) (**G**), lung adenocarcinoma (LUAD) (**H**), and ocular melanomas (UVM) (**I**).

**Figure 6:**
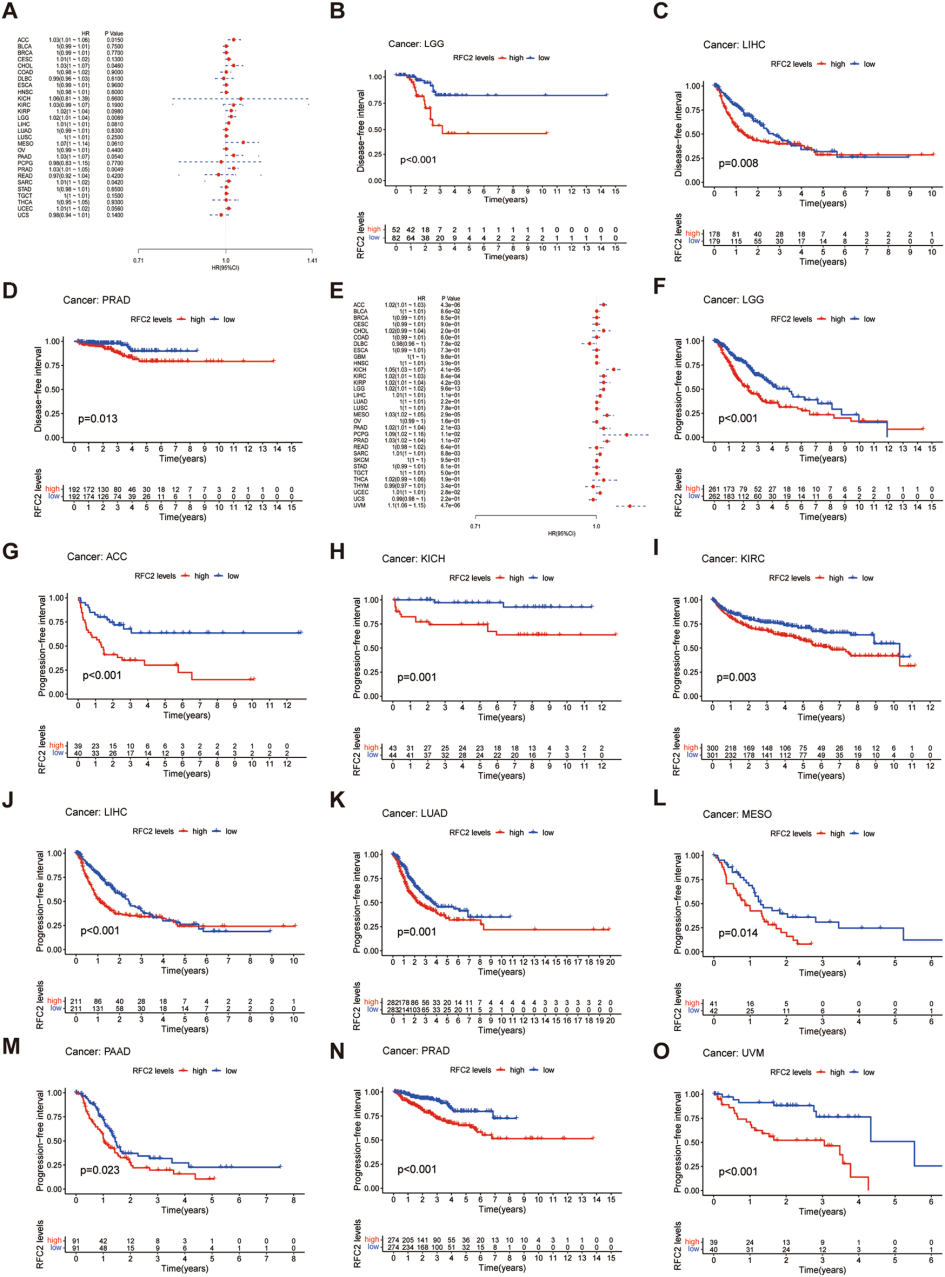
Associations of RFC2 expression with disease-free interval (DFI) and progression-free interval (PFI) across cancers in TCGA cohort. (**A**) Forest plot showing the correlation of RFC2 expression with DFI in 33 types of cancer. (**B**–**D**) Kaplan–Meier survival analyses displaying the relationships of RFC2 expression with DFI in different cancer types, including diffuse lower-grade gliomas (LGG) (**B**), liver hepatocellular carcinoma (LIHC) (**C**), and prostate Cancer (PRAD) (**D**). (**E**) Forest plot showing the correlation of RFC2 expression with PFI in 33 types of cancer. (**F**–**O**) Kaplan–Meier survival analyses displaying the relationships of RFC2 expression with PFI in different cancer types, including LGG (**F**), adrenocortical carcinoma (ACC) (**G**), kidney chromophobe (KICH) (**H**), kidney renal clear cell carcinoma (KIRC) (**I**), LIHC (**J**), lung adenocarcinoma (LUAD) (**K**), mesothelioma (MESO) (**L**), pancreatic cancer (PAAD) (**M**), prostate adenocarcinoma (PRAD) (**N**), and ocular melanomas (UVM) (**O**).

Moreover, DSS analysis displayed that RFC2 high expression was correlated with poor prognosis in ACC, BLCA, GBM, KICH, KIRC, KIRP, LGG, LIHC, LUAD, MESO, PAAD, PRAD, UCEC, and UVM (Fig. 5A). Then Kaplan–Meier survival analysis demonstrated that high levels of RFC2 were significantly related to a poorer DSS in GBM, LGG, ACC, KICH, KIRC, LIHC, LUAD, and UVM (*P* = 0.005, *P* < 0.001, *P* < 0.001, *P* < 0.001, *P* = 0.029, *P* = 0.036, *P* = 0.007, and *P* < 0.001 respectively) (Fig. 5B–I). Further for DFI analysis, the *Cox* proportional hazards model indicated obviously associations between highly expressed RFC2 and poor DFI in ACC, CHOL, LGG, PRAD, and sarcoma (SARC) (Fig. 6A). In addition, patients in RFC2 high levels groups in LGG, LIHC, and PRAD had a reduced DFI through Kaplan–Meier survival analysis (*P* < 0.001, *P* = 0.008, and *P* = 0.013 respectively) (Fig. 6B–D). Additionally, the *Cox* proportional hazards model of PFI revealed that the elevated expression of RFC2 predicted a poor PFI in ACC, KICH, KIRC, KIRP, LGG, MESO, PAAD, pheochromocytoma & paraganglioma (PCPG), PRAD, SARC, UCEC, and UVM (Fig. 6E). The results of Kaplan–Meier survival analysis found that the PFI was remarkably decreased in patients with highly expressed RFC2 in LGG, ACC, KICH, KIRC, LIHC, LUAD, MESO, PAAD, PRAD, and UVM (*P* < 0.001, *P* < 0.001, *P* = 0.001, *P* = 0.003, *P* < 0.001, *P* = 0.001, *P* = 0.014, *P* = 0.023, *P* < 0.001, and *P* < 0.001 respectively) (Fig. 6F–O). Taken together, these results indicated that RFC2 served as a powerful prognostic biomarker in multiple cancer types, especially in LGG.

### The associations between RFC2 expression and tumor mutation burden, tumor microsatellite instability, and mismatch repair genes in LGG and other cancer types

**Figure 7:**
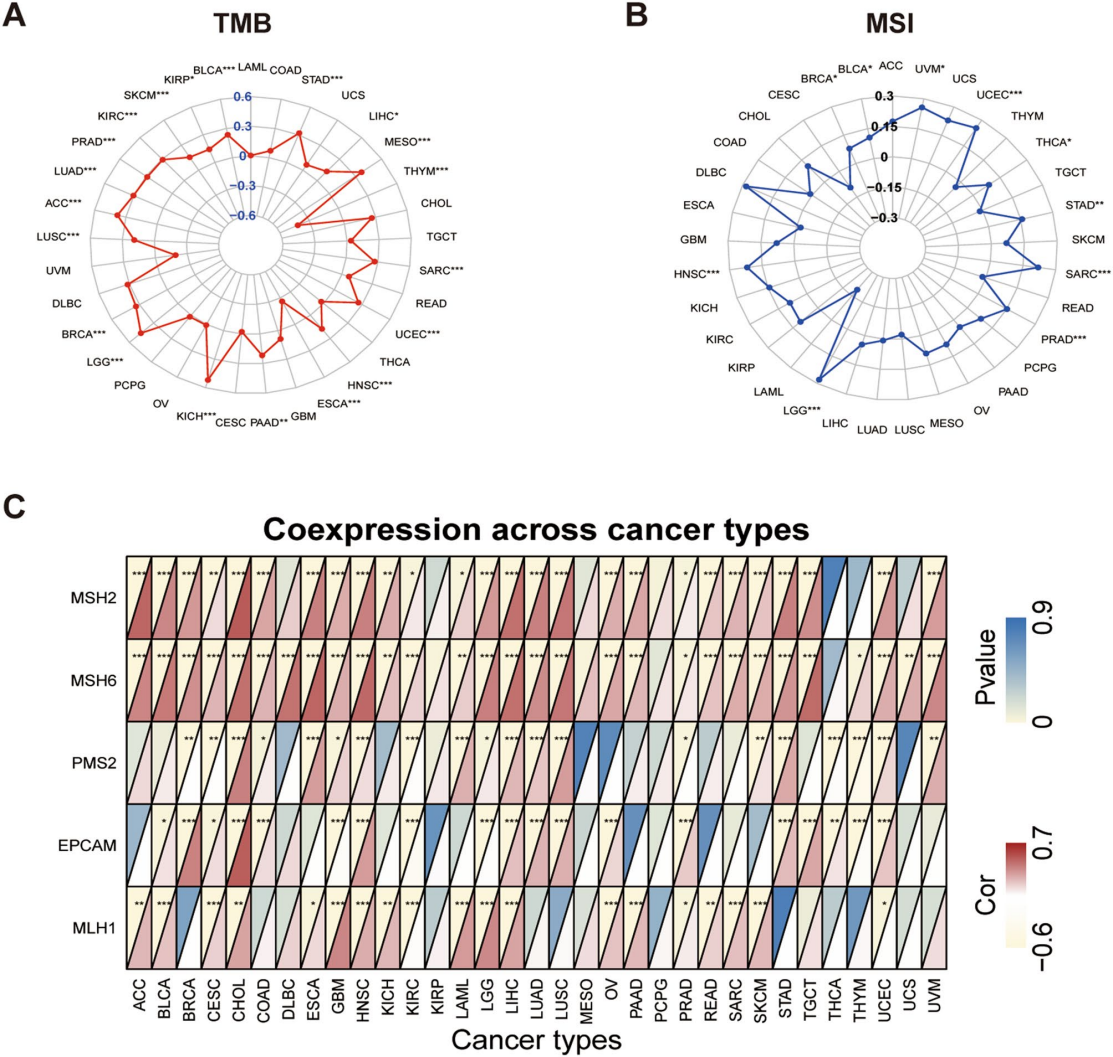
Associations between RFC2 expression and tumor mutation burden (TMB), tumor microsatellite instability (MSI), and mismatch repair (MMR) genes in pan-cancer. (**A**) Radar chart displaying the association between RFC2 expression and TMB. Numbers in blue text represent the correlation coefficient. (**B**) Radar chart displaying the association between RFC2 expression and MSI. Numbers in black text represent the correlation coefficient. (**C**) Heatmap illustrating the relationship between RFC2 expression and MMR genes. For each pair, the top left triangle represents the P-value, and the bottom right triangle represents the correlation coefficient. (*P < 0.05, **P < 0.01, ***P < 0.001).

To evaluate the potential predictive effect of RFC2 on the sensitivity to immune checkpoint inhibitors, we analyzed the associations between RFC2 expression and TMB, tumor MSI, and MMR genes in LGG and other cancer types. As shown in Fig. 7A, the expression of RFC2 was positively correlated with the TMB of 18 types of cancers, including LGG, BRCA, LUSC, ACC, LUAD, PRAD, KIRC, SKCM, KIRP, BLCA, STAD, LIHC, MESO, SARC, UCEC, HNSC, PAAD, and KICH, while its expression was negatively related with the TMB of THYM and ESCA. Further, RFC2 expression was positively correlated with the MSI in LGG and other 9 types of cancers, including HNSC, BRCA, BLCA, UVM, UCEC, THCA, STAD, SARC, and PRAD (Fig. 7B). Then we estimated the possible relationships of RFC2 expression with the expression of MMR genes, including MutS homologous 2 (MSH2), MSH6, postmeiotic segregation increased 2 (PMS2), epithelial cell adhesion molecule (EPCAM), and MutL homologous gene (MLH1). And the results showed that RFC2 levels had a clearly positive correlation with MMR gene expression in LGG and other most tumors, except KIRP, MESO, and PCPG (Fig. 7C). Therefore, these results indicated that RFC2 acted as a reliable indicator to predict the sensitivity of LGG and other tumors to immune checkpoint inhibitors.

### The correlations of RFC2 levels with immune cell infiltration and immune checkpoints

**Figure 8:**
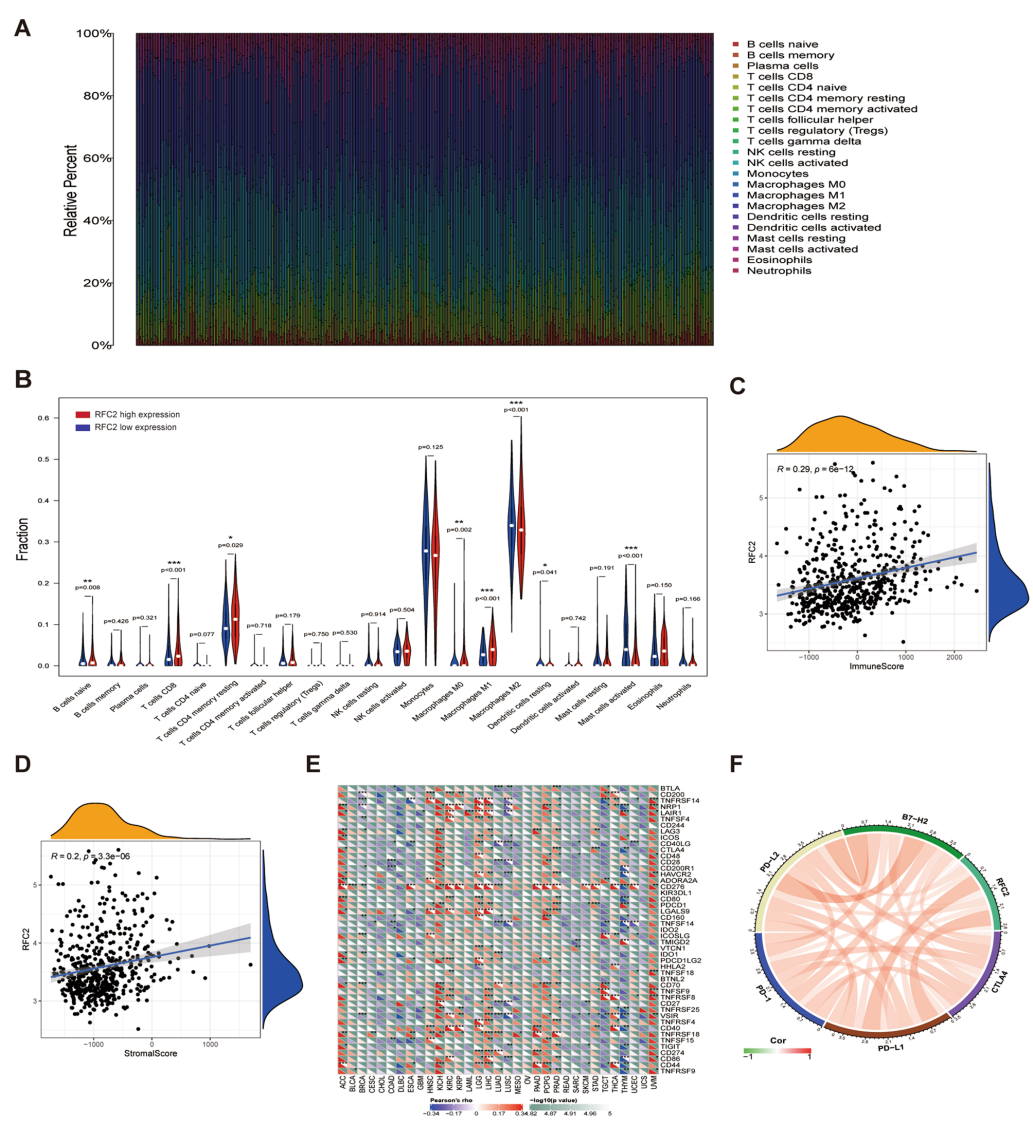
The correlations of RFC2 levels with immune cell infiltration and immune checkpoints. (**A**) The proportions of 22 immune cell types in each sample of LGG from TCGA cohort were estimated by conducting CIBERSORT algorithm. (**B**) Correlations of RFC2 expression with differential immune cell types expression in LGG. (**C**,**D**) Associations of RFC2 expression with immune score (**C**) and stromal score (**D**) were examined by performing ESTIMATE algorithm. (**E**) Heatmap illustrating the relationship between RFC2 expression and immune checkpoint genes expression across 33 types of cancer. For each pair, the bottom left triangle represents the correlation coefficient, and the top right triangle represents the P-value. (**F**) Association between RFC2 and immune checkpoints genes in LGG. (*P < 0.05, **P < 0.01, ***P < 0.001).

To further investigate the underlying functions of RFC2 in immune cell infiltration in LGG, we performed the CIBERSORT algorithm to examine the proportions of 22 immune cell types in each sample of LGG (Fig. 8A). The results showed that the proportions of the naïve B cells (*P* = 0.008), CD8^+^ T cells (*P* < 0.001), resting memory CD4^+^ T cells (*P* = 0.029), M0 macrophages (*P* = 0.002), and M1 macrophages (*P* < 0.001) in RFC2 high expression group were apparently higher than that in RFC2 low expression group, whereas the fractions of M2 macrophages (*P* < 0.001), resting dendritic cells (*P* = 0.041), and activated mast cells (*P* < 0.001) was relatively much lower in RFC2 high expression group (Fig. 8B). Then we adopted the ESTIMATE algorithm to calculate the stromal and immune scores in LGG and found that RFC2 expression was significantly positively associated with both immune scores (R = 0.29, *P* < 0.001) and stromal scores (R = 0.2, *P* < 0.001) in LGG (Fig. 8C,D). Further, we proceeded to evaluate the associations between RFC2 expression and immune checkpoint genes expression in pan-cancer. As displayed in Fig. 8E, the heatmap showed that in multiple cancers, except BLCA, CESC, CHOL, DLBC, GBM, LAML, MESO, OV, READ, and UCS, the robust correlations existed between the expression of RFC2 and the expression of recognized immune checkpoint genes, including B- and T-lymphocyte attenuator (BTLA), CD200, tumor necrosis factor receptor superfamily 14 (TNFRSF14), neuropilin 1 (NRP1), leukocyte-associated immunoglobulin-like receptor 1 (LAIR1), lymphocyte activation gene 3 (LAG3), inducible T cell costimulator (ICOS), CD40 ligand (CD40LG), CTLA4, CD48, CD28, CD200 receptor 1 (CD200R1), hepatitis A virus cellular receptor 2 (HAVCR2), CD276, CD80, PDCD1, LGALS9, CD160, TNFSF14, IDO1, programmed cell death 1 ligand 2 (PDCD1LG2), CD70, TNFSF9, TNFRSF8, CD27, CD40, TNFRSF18, CD274, and CD44. Additionally, we further explored the relationships between RFC2 and immune checkpoints members in LGG and found that the expression levels of RFC2 were significantly correlated with the pivotal immune checkpoint genes, including PD-1, PD-L1, PD-L2, B7-H2, and CTLA4 (Fig. 8F and Supplementary Table S1), indicating the potential value of RFC2 for immunotherapy response in LGG.

### The functional enrichment analyses of RFC2 in LGG

**Figure 9:**
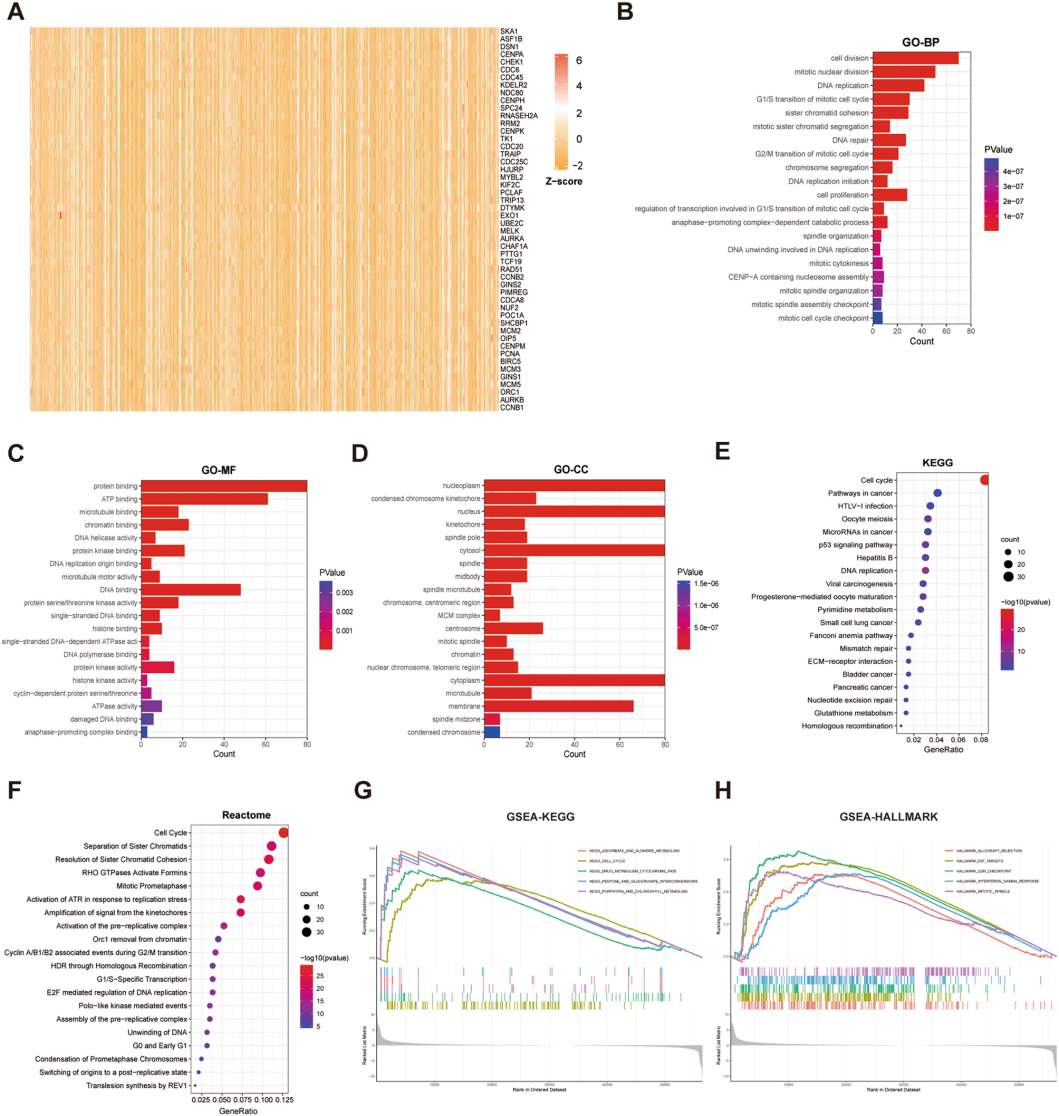
The functional enrichment analyses of RFC2 in LGG. (**A**) Heatmap showing the top 50 genes most positively related to RFC2. (**B**–**D**) The top 20 Gene Ontology (GO) terms related to RFC2 in biological processes (BP) categories (**B**), molecular function (MF) categories (**C**), and cell component (CC) categories (**D**) respectively. (**E**) The top 20 Kyoto Encyclopedia of Genes and Genomes (KEGG) pathways correlated with RFC2. (**F**) The top 20 Reactome pathways correlated with RFC2. (**G**) GSEA results showing the top 5 KEGG pathways associated with RFC2. (**H**) GSEA results showing the top 5 HALLMARK pathways associated with RFC2.

To further explore the functions and pathways related to RFC2, we performed a correlation analysis between RFC2 gene and other genes in LGG by using TCGA data. The 300 genes that positively related to RFC2 were identified for enrichment analysis, and the top 50 genes were screened to produce a heatmap (Fig. 9A). Then the clusterProfiler R package was used to explore the potential function pathways associated with RFC2. The GO analysis revealed that RFC2 was mostly correlated with the functional pathways of cell proliferation, including cell division, mitotic nuclear division, DNA replication, and cell cycle checkpoints (Fig. 9B–D). Furthermore, The KEGG pathway analysis showed that RFC2 was mainly enriched in cell cycle, pathways in cancer, HTLV-I infection, oocyte meiosis, and microRNAs in cancer terms (Fig. 9E). The Reactome analysis demonstrated the significant enrichment of RFC2 in cell cycle, separation of sister chromatids, resolution of sister chromatid cohesion, RHO GTPases activate formins, and mitotic prometaphase terms (Fig. 9F). In addition, we next conducted GSEA by using KEGG and HALLMARK datasets. The results of KEGG by GSEA revealed that the ascorbate and aldarate metabolism, cell cycle, drug metabolism cytochrome p450, pentose and glucuronate interconversions, and porphyrin and chlorophyll metabolism pathways were evidently enriched (Fig. 9G). Moreover, allograft rejection, E2F targets, G2M checkpoint, interferon gamma response, and mitotic spindle pathways were remarkably enriched by HALLMARK pathway analysis (Fig. 9H). Taken together, these results indicated that RFC2 was closely associated with many oncogenic pathways involved in carcinogenesis and tumor progression in LGG.

### RFC2 knockdown inhibited the progression of LGG cells

**Figure 10:**
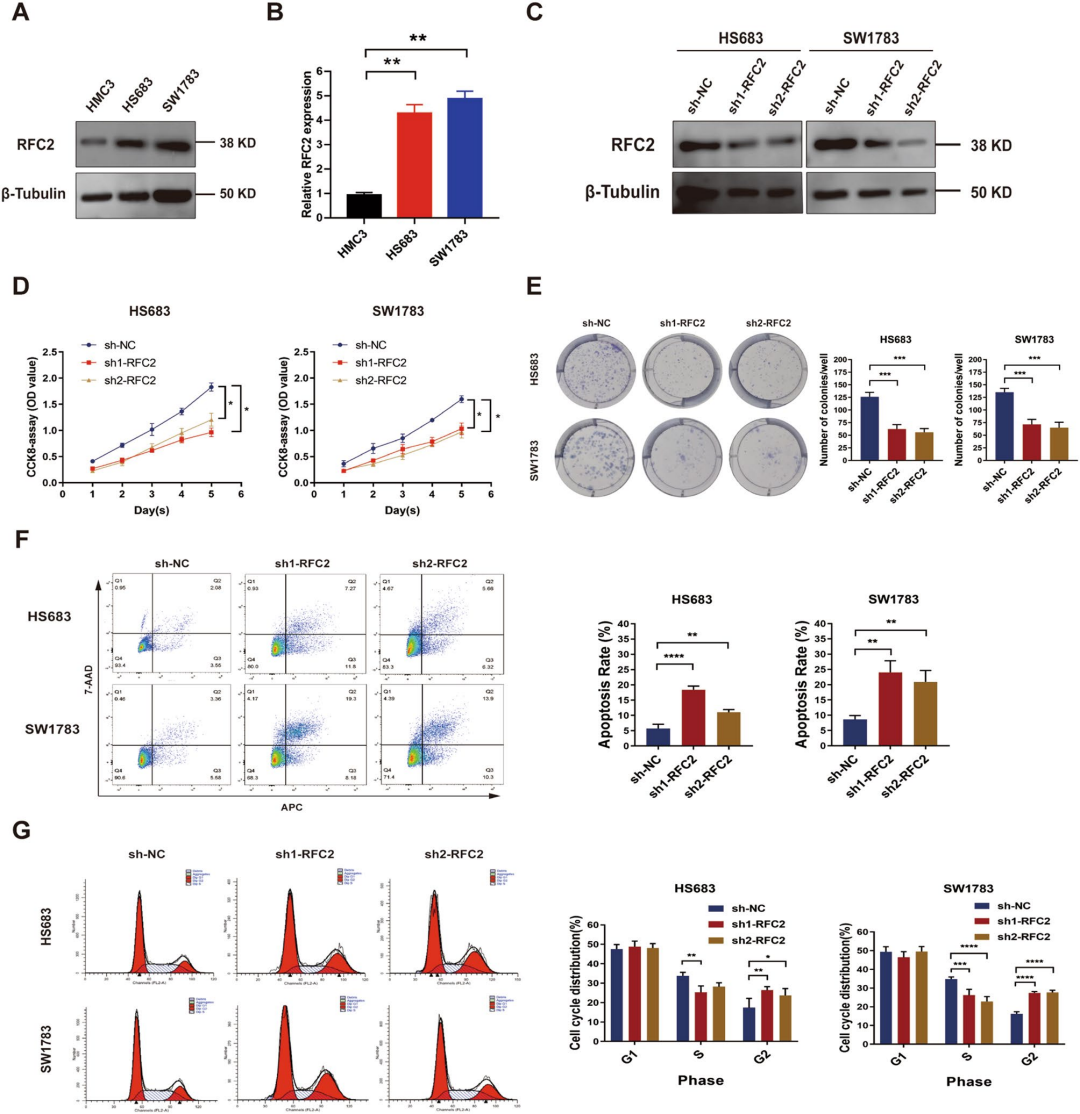
RFC2 knockdown inhibited the progression of LGG cells. (**A**,**B**) The relative expression levels of RFC2 in LGG cells (HS683 and SW1783) and microglia (HMC3) were estimated by Western blot analysis (**A**) and qRT-PCR analysis (**B**). (**C**) The relative expression levels of RFC2 in LGG cells transfected with sh1-RFC2, sh2-RFC2, and sh-NC were evaluated by Western blot analysis. (**D**) CCK-8 assay was conducted to assess the proliferation of LGG cells transfected with sh1-RFC2, sh2-RFC2, and sh-NC. (**E**) Colony formation assay was performed to evaluate the colony formation capability of LGG cells transfected with sh1-RFC2, sh2-RFC2, and sh-NC. (**F**) Apoptotic rate of LGG cells transfected with sh1-RFC2, sh2-RFC2, and sh-NC was detected by using flow cytometry. (**G**) Cell cycle distribution of LGG cells transfected with sh1-RFC2, sh2-RFC2, and sh-NC was examined by using flow cytometry. (*P < 0.05, **P < 0.01, ***P < 0.001, ****P < 0.0001).

Next, we verified the effect of RFC2 on the progression of LGG cells. Western blot and qRT-PCR analysis showed that both the mRNA and protein levels of RFC2 in LGG cells, including HS683 and SW1783 cell lines, were apparently higher than those in human microglia HMC3 cells (Fig. 10A,B and Supplementary Fig. S1). We next suppressed the expression of RFC2 in LGG cells and tested the knockdown efficiency by performing Western blot and qRT-PCR analysis (Fig. 10C and Supplementary Fig. S2). We found that knockdown of RFC2 significantly restrained cell proliferation and colony formation ability in LGG cells (Fig. 10D,E). Furthermore, the flow cytometry analysis showed that RFC2 knockdown remarkably increased apoptosis rates of LGG cells (Fig. 10F). Then, we examined the impact of RFC2 depletion on cell cycle distribution of LGG. The results showed that a much higher proportion of LGG cells with RFC2 stable knockdown were arrested in G2 phase (Fig. 10G), compared to their corresponding control cells. Thus, these results strongly suggested that RFC2 acted as an oncogene to promote the malignant progression of LGG cells.

### RFC2 expression correlated with clinicopathological features in our LGG samples

**Figure 11:**
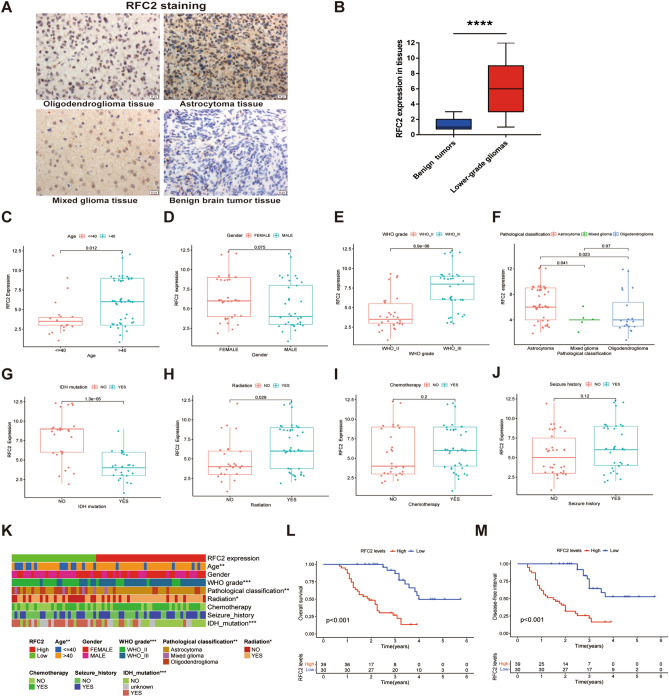
RFC2 expression correlated with clinicopathological features in our LGG samples. (**A**) The immunohistochemistry images showing the relative expression of RFC2 in LGG tissues and benign brain tumor tissues (Original magnification, × 400). (**B**) RFC2 expression levels in LGG tissues (n = 69) and benign brain tumor tissues (n = 10) were evaluated by immunohistochemistry analysis. (**C**–**J**) The associations of RFC2 expression with clinicopathological factors, including age (**C**), gender (**D**), WHO grade (**E**), pathological classification (**F**), IDH1 mutation status (**G**), radiation therapy history (**H**), chemotherapy history (**I**), and seizure history (**J**). (**K**) Heatmap displaying the correlation between RFC2 expression and clinicopathological features in our LGG patients. (**L**,**M**) Kaplan–Meier survival analyses showing the relationships of RFC2 expression with overall survival (OS) (**L**) and disease-free survival (DFS) (**M**) in LGG patients. (*P < 0.05, **P < 0.01, ***P < 0.001, ****P < 0.0001).

To further confirm the potential oncogenic role of RFC2 in LGG, we examined RFC2 expression and performed a clinical correlation analysis of RFC2 in our LGG samples, which contained 69 LGG tissues and 10 benign brain tumor tissues. The immunohistochemistry staining results showed that RFC2 was primarily localized in the nucleus, and RFC2 was significantly highly expressed in LGG tissues than that in benign brain tumor tissues (Fig. 11A,B). Further, we continued to verify the correlation between RFC2 expression and the clinicopathological features in our LGG samples. We noticed that RFC2 was highly expressed in the > 40 years age group than that in the ≤ 40 years age group (*P* = 0.012) (Fig. 11C). However, no significant correlation was observed between RFC2 expression and gender of LGG patients (*P* = 0.075) (Fig. 11D). Moreover, RFC2 expression was obviously increased in LGG samples with WHO III grade (*P* < 0.001), astrocytoma (*P* = 0.041 and *P* = 0.023), and IDH1 wild-type (*P* < 0.001) (Fig. 11E–G). Additionally, the differential expression levels of RFC2 were also associated with LGG patients who received radiotherapy (*P* = 0.029) (Fig. 11H), whereas there were no marked associations between RFC2 expression and the history of chemotherapy (*P* = 0.2) and seizure (*P* = 0.12) in LGG patients (Fig. 11I,J). Interestingly, these results were consistent with the analysis results of the LGG cohort from TCGA database (Fig. 11K). We next investigated the effect of RFC2 on the prognosis of our LGG samples, and Kaplan–Meier survival analysis showed that high expression levels of RFC2 were significantly related to poor OS (*P* < 0.001) and disease-free survival (DFS) (*P* < 0.001) in LGG patients (Fig. 11L,M). Therefore, these results demonstrated that the aberrant elevated expression of RFC2 was closely correlated with increased tumor malignancy and unfavorable prognosis in LGG patients.

## Discussion

Despite the rapid advancement in standard comprehensive treatments, including maximum surgical resection and adjuvant chemoradiotherapy, a vast subset of LGG patients have inevitably suffered from recurrence or malignant progression due to the highly invasive nature of LGG^20,21^. Furthermore, the distinct immunosuppressive microenvironment of LGG constitutes a considerable barrier for attenuating the anti-tumoral immune responses in LGG^22^. Consequently, the identification of appropriate predictive and therapeutic targets for improving the outcomes of LGG patients has far-reaching significance. The RFC2 gene, encoding the third-largest subunit of the RFC complex, plays a critical role in DNA replication and repair in eukaryotic cells^23^. Previous limited studies hinted that RFC2 exerted a potential carcinogenic role in several cancer types, including CRC^18^, HCC^19^, and ESCC^24^. However, the specific functions of RFC2 in gliomas have rarely been reported. In the present study, we confirmed that RFC2 was generally up-regulated at both the mRNA and protein levels in a broad set of human cancers, including LGG. Besides, the elevated expression of RFC2 was closely related to the clinicopathological features of LGG patients from TCGA dataset. Moreover, the *Cox* proportional hazards model and Kaplan–Meier survival analysis revealed that the high expression levels of RFC2 strongly predicted the poor prognosis in multiple cancer types, such as GBM, LGG, ACC, KICH, KIRC, LUAD, and UVM. In contrast, its high expression was associated with favorable survival in CESC. The above results indicated that RFC2 served as a powerful prognosis biomarker in multiple cancers, especially in LGG.

TMB represents a reliable estimation for tumor neoantigen burden, which is widely proved to act as a sensitive and specific indicator to predict the efficacy of immunotherapy in various types of cancer^25^. Previous investigations demonstrated that high TMB was closely related to greater response rates to ICB therapy across different malignancies, including NSCLC^26^, BRCA^27^, renal cell carcinoma^28^, and colorectal cancer^29^. MSI, a molecule phenotype arising from a deficiency in the DNA mismatch repair system, is considered as another actionable biomarker for anti-tumor immunotherapy^30^. An increasing number of clinical trials revealed that patients with high levels of MSI (MSI-H) achieved improved outcomes from the treatment of ICB compared with those with the patterns of low levels of MSI (MSI-L) or stable microsatellite (MSS)^31–33^. Our present study verified that the expression levels of RFC2 were positively correlated with TMB in 18 cancer types and negatively related with that in THYM and ESCA. Besides, RFC2 expression was also closely associated with MSI in LGG and another 9 types of cancers. In addition, we also noted a positive correlation between RFC2 levels and MMR genes in most cancers, including LGG. Taken together, these findings suggested that RFC2 levels might be used to assess the TBM levels and MSI status, thereby being established in combination with them as new thresholds to predict the anti-tumor immune response of ICB in LGG and other cancer types.

TME refers to a highly complex and dynamic cellular environment, which is composed of various cell types, including cancer cells, stromal cells, fibroblasts, endothelial cells, and immune cells^34^. Growing evidence uncovered that the sustained crosstalk between cancer cells and immune cells in TME ultimately modulated cancer progression, migration, and treatment resistance^35^. In particular, the glioma microenvironment is generally featured as an immunosuppressive status since been tightly regulated by the interaction of its unique compositions, including neoplastic glioma cells, macrophages, microglia, tumor-infiltrating lymphocytes (TILs), and part of the blood–brain barrier (BBB), which also presents an enormous challenge for immunotherapy efficacy^36^. By conducting the CIBERSORT algorithm, we first calculated the abundance ratios of 22 immune cell types in each LGG sample from TCGA. Then we evaluated the correlations between RFC2 expression and the infiltration of immune cells in LGG. We noted that RFC2 high expression group had obviously higher proportions of the naïve B cells, CD8^+^ T cells, resting memory CD4^+^ T cells, M0 macrophages, and M1 macrophages than RFC2 low expression group, yet relative lower fractions of M2 macrophages, resting dendritic cells, and activated mast cells were observed in RFC2 high expression group. Furthermore, as expected, we found clear positive associations between RFC2 expression and both immune scores and stromal scores in LGG by performing the ESTIMATE algorithm. In addition, our work also clarified that RFC2 had robust co-expression relationships with the recognized immune checkpoint genes, such as PD-1, PD-L1, PD-L2, B7-H2, and CTLA4 in LGG. Therefore, these results implied that RFC2 played a potential regulatory role in activating anti-tumor immune responses in LGG, and proposed a new target for LGG immunotherapy.

Moreover, we next conducted the functional enrichment analyses of RFC2 in LGG and found that RFC2 was mainly enriched in cancer-related pathways, including cell division, mitotic nuclear division, DNA replication, cell cycle checkpoints, and drug metabolism-related pathways. We further preliminarily validated the role of RFC2 in LGG progression by performing functional assays in vitro. And we found that RFC2 attenuation inhibited proliferation and promoted cell apoptosis and G2 phase arrest in LGG cells. Our results highlighted a potentially essential function of RFC2 in modulating the progression in LGG. These data are in line with previously reported studies, illustrating that RFC2 was strongly involved in cell proliferation, DNA repair, and chemoresistance^37–39^. Additionally, we then detected RFC2 expression and performed a clinical correlation analysis of RFC2 in our collected LGG samples. Our results showed that RFC2 was highly expressed in LGG tissues. Besides, the expression levels of RFC2 were closely correlated with the clinicopathological features of LGG, including age, WHO grade, pathological type, IDH-1 status, and radiotherapy history. Further, RFC2 overexpression was also related to a poor prognosis in patients with LGG. These results were consistent with the analyses of the LGG cohort from TCGA database, confirming a potentially oncogenic role of RFC2 in LGG.

In conclusion, our comprehensive pan-cancer analysis discovered for the first time that RFC2 was generally highly expressed in multiple cancer types, including LGG. Besides, its elevated expression was closely associated with the clinicopathological features of LGG. Further, RFC2 acted as an independent prognostic factor for LGG and other malignancies. Our study demonstrated tightly relationships between RFC2 expression and TMB and MSI across various cancers. Moreover, the expression levels of RFC2 were also correlated with the infiltration of immune cells and immune checkpoint genes in LGG. Thus, our findings indicated that RFC2 could serve as a valuable prognostic biomarker for LGG, and provided a novel therapeutic target for LGG immunotherapy.

## Methods

### Data collection and processing

The RNA sequencing data, the relevant clinical data, as well as the somatic mutation data of 33 types of cancer were downloaded from TCGA (http://cancergenome.nih.gov) by using UCSC Xena (https://xena.ucsc.edu/). Human gene expression data from 54 tissue sites were acquired from GTEx (http://commonfund.nih.gov/GTEx/). The gene expression data of RFC2 from these datasets were extracted by using Strawberry Perl (Version 5.32.1.1, http://strawberryperl.com/). Furthermore, 69 paraffin-embedded LGG tissues were obtained from patients with pathological confirmation who received standard surgery and chemoradiotherapy at the First Affiliated Hospital of Xi'an Jiaotong University from 2013 to 2018. Besides, 10 paraffin-embedded benign brain tumor tissues were acquired to represent the control group. Our research was approved by the Ethics Committee of the First Affiliated Hospital of Xi’an Jiaotong University (Approval number: XJTU1AF2018LSK-108). And all of the patients had signed the written informed consent. All assays were conducted in accordance with relevant guidelines and regulations, which were consistent with the Declaration of Helsinki regulations. The detailed clinical data of our LGG samples are supplied in Supplementary Tables S2, S3.

### RFC2 expression profiling analysis

Oncomine database (https://www.oncomine.org/resource/main.html), which integrated the publicly available tumor microarray datasets, was used to evaluate RFC2 expression in various types of cancer, with the fold change as 1.5 and P-value cutoff of 0.05 as significant. Furthermore, we employed the TIMER database (https://cistrome.shinyapps.io/timer/) to analyze the differential expression levels of RFC2 in tumor tissues and normal tissues in multiple cancers. In addition, RFC2 expression was compared between the cancer samples from TCGA and the normal samples from both TCGA and GTEx. Moreover, HPA (http://www.proteinatlas.org/) and the GEPIA (http://gepia.cancer-pku.cn/index.html) were conducted to further evaluate RFC2 protein expression levels in six cancer tissues and matched normal adjacent tissues, including GBM, LGG, CESC, COAD, LIHC, and LUSC.

### Analysis of the correlation between RFC2 and the clinicopathological features

The clinical phenotypes, including patient age, gender, WHO grade, pathological classification, IDH1 mutation status, radiation therapy history, chemotherapy history, and seizure history, were extracted from the LGG cohort of TCGA and our LGG cohort to investigate their correlation with RFC2 expression. The R packages “limma” and “ggpubr” were conducted to analyze the relationships between RFC2 expression and clinicopathological features in LGG, and *P* < 0.05 was considered as significant.

### Survival analysis

The survival data of 33 types of cancer, which contained the indicators of OS, DSS, DFI, and PFI, were downloaded from TCGA database. The Kaplan–Meier analysis and log-rank test were then conducted for survival analysis. The R packages “survival” and “survminer” were used to plot the survival curves. Furthermore, the *Cox* proportional hazards model was performed to evaluate the association between RFC2 expression and survival of pan-cancer by using the R packages of “survival” and “forestplot”.

### Correlation analyses of RFC2 expression with TMB, MSI, and MMR genes

TMB served as a quantifiable immune-response measure that reflected the number of mutations per megabase (Mb) of the tumor cell genome^40^. The TMB scores were calculated by using a Perl script based on the somatic mutation data of 33 types of cancer downloaded from TCGA database. Then the association between RFC2 expression and TMB of pan-cancer was determined. MSI was utilized as an indicator to detect the instability of the tumor genome, which was often used to evaluate the responsiveness of immunotherapy^30^. We calculated the MSI scores for each cancer from TCGA, and then performed correlation analysis between MSI and RFC2 expression. Additionally, we next extracted the expression profiles of the MMR genes, which containing MSH2, MSH6, PMS2, EPCAM, and MLH1, from TCGA pan-cancer data, and subsequently analyzed the association between the expression levels of MMR genes and that of RFC2. The R packages of “reshape2” and “RColorBrewer” were used to generate the heatmap to display gene correlations.

### Association analyses of RFC2 expression with immune cell infiltration

CIBERSORT algorithm was conducted to calculate the proportions of 22 types of TICs in each sample of LGG. Then the correlations between RFC2 expression and each TIC type in LGG were evaluated and visualized by using the R packages of “limma” and "vioplot" (*P* < 0.05 as significant).

### Evaluation of immune score and stromal score

ESTIMATE algorithm was utilized to compute the immune score and stromal score for each sample of LGG, then the associations between RFC2 expression and these two scores were analyzed by conducting the R packages of “limma” and “estimate”.

### Correlation analyses of RFC2 with immune checkpoint genes

A co-expression analysis between RFC2 expression and immune checkpoint genes was assessed and visualized by using the R packages of “limma”, “reshape2”, and “RColorBreyer”.

### Correlation and functional enrichment analyses

The Pearson co-expression correlation analysis was conducted to estimate the relationship between RFC2 expression and other mRNAs in LGG by using TCGA LGG data. Then the top 300 genes, which were most positively associated with RFC2 expression, were screened for further enrichment analysis to explore the functional role of RFC2 in LGG. Subsequently, GO function enrichment analysis, KEGG pathway enrichment analysis, as well as Reactome pathway enrichment analysis were performed by using the clusterProfiler R package. Furthermore, GSEA was applied to further examine the biological functions of RFC2 in LGG. KEGG and HALLMARK gene sets were downloaded from the GSEA website (http://www.gsea-msigdb.org/gsea/index.jsp). Then GSEA functional analyses of these two gene sets were performed by using the R packages of “limma”, “org.Hs.eg.db”, “clusterProfiler”, and “enrichplot”.

### Immunohistochemistry analysis

The paraffin-embedded tissue sections, including LGG and benign brain tumor tissue sections, were dewaxed and then hydrated. Then the sections were heated with sodium citrate buffer (JISSKANG Biotechnology, Qingdao, China) for antigenic repair for 30 min. After incubated with 3% H_2_O_2_ at room temperature for 10 min, the sections were then blocked with a blocking buffer of 10% goat serum for 15 min at room temperature. Subsequently, the sections were incubated overnight at 4 °C with RFC2 (1:200, NBP1-89341, Novus, USA) primary antibody. Then the tissue sections were incubated with the HRP-labeled secondary rabbit antibody and then with the HRP-labeled streptavidin reagent. Next, the diaminobenzidine (DAB) (Zhongshan Golden Bridge, Beijing, China) was used to stain the tissue sections. Finally, the staining sections were photographed and analyzed by applying microscopy (Leica, Germany). The eventual immunoreactive score of RFC2 in each tissue section was determined by calculating the product of the percentage of positive cells and the staining intensity.

### Cell culture and transfection

Human glioma HS683 cell line and human microglia HMC3 cell line was purchased from Procell Life Science & Technology Co., Ltd. (Wuhan, China). And human glioma SW1783 cell line was obtained from Shanghai Yaji Biological Technology Co., Ltd (Shanghai, China). The cell lines were cultured in a DMEM medium (Procell, Wuhan, China) with 10% fetal bovine serum (FBS; Gibco, Rockville, MD) and 1% penicillin‐streptomycin (Procell, Wuhan, China) at 37 °C in a 5% CO_2_ incubator. To acquire RFC2 stable knockdown LGG cells, the specific RFC2 short hairpin RNA lentivirus (sh1-RFC2 and sh2-RFC2) and their corresponding negative control shRNA lentivirus (sh-NC), which both of them were synthesized by Genechem (Shanghai, China), were transfected into LGG cells by using Lipofectamine 3000 reagent (Invitrogen). Then, puromycin (5 μg/ml) was added to the medium to screen the stably transfected cells. The transfection efficiency of LGG cells was then verified by performing Western blot and qRT-PCR analysis.

### qRT-PCR assay

The total RNA was extracted from cell lines by using Trizol reagent (Invitrogen, Carlsbad, CA, USA). The cDNA was synthesized and generated by using the First-strand cDNA synthesis kit (Tiangen Biotech, Beijing, China). Subsequently, the expression levels of RFC2 were detected by performing qRT-PCR with a SYBR^®^ Premix Dimer Eraser kit (Takara Shiga, Japan). GAPDH was used as the inner reference. The expression levels were calculated by a 2-ΔΔCt method. The primers used for examining RFC2 and GAPDH levels were as follows: RFC2-Forward: 5ʹ-GTGAGCAGGCTAGAGGTCTTT-3ʹ; RFC2-Reverse: 5ʹ-TGAGTTCCAACATGGCATCTTTG-3ʹ; GAPDH-Forward: 5ʹ-GTGAAGGTCGGAGTCAAC-3ʹ; GAPDH-Reverse: 5ʹ-GTTGAGGTCAATGAAGGG-3ʹ.

### Western blot analysis

The total proteins from HS683, SW1783, and HMC3 cells were extracted by using radioimmunoprecipitation (RIPA) lysis buffer (Sigma Aldrich, Cambridge, MA), and then quantified by using BCA Protein Assay Kit (Roche, Switzerland). Then, sodium dodecyl sulfate–polyacrylamide gel electrophoresis (SDS-PAGE) was used to separate proteins. Proteins were transferred onto polyvinylidene fluoride (PVDF) membranes (Millipore, USA), which were then incubated overnight at 4 °C with primary antibodies, including RFC2 (1:1000, 10410-1-AP, Proteintech, USA) and β-Tubulin (1:1000, 10094-1-AP, Proteintech, USA). Subsequently, the membranes were then incubated with the secondary antibody (1:2000, Beyotime, Shanghai, China) labeled with horseradish peroxidase for 1 h at room temperature. Then, the protein bands were visualized by using the electrochemiluminescence reagent kit (Millipore, USA).

### Cell counting kit‑8 (CCK‑8) assay

CCK-8 kit (AbMole, USA) was used to evaluate the proliferation of LGG cells according to the manufacturer’s protocol. LGG cells were plated into a 96-well plate with a concentration of around 1 × 10^3^ per well. Then, cells were cultured at 5% CO_2_ and 37 °C for 24 h, 48 h, 72 h, 96 h, and 120 h respectively. Each well was then supplemented with 10 μL CCK-8 reaction solution followed by 2 h incubation. Then, the optical density (OD) values at 450 nm were recorded.

### Colony formation assay

LGG cells were seeded into 6-well plates with a concentration of 500 cells per well. Then cells were incubated in a 5% CO_2_ and 37 °C incubator for 2 weeks. Next, cultures were terminated by fixation in 4% paraformaldehyde for 30 min and then stained with Giemsa for 20 min. Finally, the colonies containing 50 or more cells were counted.

### Cell apoptosis assay

Annexin V-APC/7-AAD apoptosis Kit (MultiSciences, Hangzhou, China) was used to detect the apoptosis rate of LGG cells. LGG cells seeded on the 6-well plates were washed with ice-cold PBS and harvested with a concentration of 5 × 10^5^ per well. Then, cells were resuspended in 1 × binding buffer and then supplemented with Annexin V-APC and 7AAD, followed by incubation in dark at room temperature for 5 min. Flow cytometry was then used to analyze the cell apoptosis rate of LGG cells.

### Cell cycle analysis

A Cell cycle staining Kit (MultiSciences, Hangzhou, China) was used for detecting cell cycle distribution of LGG cells. LGG cells were cultured in a 6-well plate and harvested with a concentration of 5 × 10^5^ per sample. Then, cells were washed with ice-cold PBS and fixed with 70% pre-cooled ethanol at 4 °C overnight. After being centrifuged and resuspended with ice-cold PBS, cells were added with DNA staining solution and permeabilization solution at room temperature in darkness for 30 min incubation. Finally, flow cytometry was used to evaluate cell cycle distribution.

### Statistical analysis

All data referring to gene expression was normalized by log2 transformation. A paired Student’s t-test was conducted to compare the differences between tumor tissues and normal tissues. One-way ANOVA was used for the comparison of multiple groups. The Kaplan–Meier survival analysis, log-rank test, and the *Cox* proportional hazards model were applied for survival analyses. The Pearson’s and Spearman’s correlation tests were performed to analyze the association between two variables. The R software (Version 4.0.5) and the GraphPad Prism 8.2 software were applied to process and visualize the statistical data involved in this study, and *P* < 0.05 was indicative of a statistically significant difference.

## Supplementary Information

Supplementary Information.
