# Supplementary material for: RFC2: a prognosis biomarker correlated with the immune signature in diffuse lower-grade gliomas
Source: Sci Rep. 2022 Feb 24;12:3122. doi: 10.1038/s41598-022-06197-5 (PMC8873322; doi:10.1038/s41598-022-06197-5)
Supplement: Supplementary file 1 — Supplementary Information. [file 41598_2022_6197_MOESM1_ESM.pdf]

Title： RFC2: A prognosis biomarker correlated with the immune signature in diffuse lower-grade gliomas

Author details：

Xu Zhao, Department of Radiation Oncology, The First Affiliated Hospital of Xi'an Jiaotong University, Xi'an, Shaanxi, 710061, China. e-mail: zx19890305@stu.xjtu.edu.cn;

Yuzhu Wang, Department of Radiation Oncology, The First Affiliated Hospital of Xi'an Jiaotong University, Xi'an, Shaanxi, 710061, China. e-mail: wyz960426@163.com;

Jing Li, Department of Radiation Oncology, The First Affiliated Hospital of Xi'an Jiaotong University, Xi'an, Shaanxi, 710061, China. e-mail: lijing8656@stu.xjtu.edu.cn;

Fengyi Qu, Department of Radiation Oncology, The First Affiliated Hospital of Xi'an Jiaotong University, Xi'an, Shaanxi, 710061, China. e-mail: vesper@stu.xjtu.edu.cn;

Xing Fu, Department of Radiation Oncology, The First Affiliated Hospital of Xi'an Jiaotong University, Xi'an, Shaanxi, 710061, China. e-mail: 1977349604@qq.com;

Siqi Liu, Department of Radiation Oncology, The First Affiliated Hospital of Xi'an Jiaotong University, Xi'an, Shaanxi, 710061, China. e-mail: 842401659@qq.com;

Xuan Wang, Department of Radiation Oncology, The First Affiliated Hospital of Xi'an Jiaotong University, Xi'an, Shaanxi, 710061, China. e-mail: 1162947000@qq.com;

Yuchen Xie, Department of Radiation Oncology, The First Affiliated Hospital of Xi'an Jiaotong University, Xi'an, Shaanxi, 710061, China. e-mail: 809585640@qq.com;

Corresponding Author: Xiaozhi Zhang, Department of Radiation Oncology, The First Affiliated Hospital of Xi'an Jiaotong University, No.277, Yanta West Road, 710061, Xi'an, Shaanxi, China. e-mail: zhangxiaozhi@xjtu.edu.cn

Supplementary Table S1. Correlation between RFC2 expression and immune checkpoint genes in LGG

Immune checkpoint genes	*Spearman correlation (R value)*	*P-value*
PD-1	0.30	4.2e-13
PD-L1	0.49	3.6e-06
PD-L2	0.29	1.1e-11
B7-H2	0.19	5.2e-05
CTLA4	0.18	3.1e-05

LGG, Diffuse lower-grade gliomas.

Supplementary Table S2. Clinical data of patients with LGG and benign brain tumor

Clinicopathological factor	*LGG* (*n*=69)	*Benign brain tumor* (*n*=10)
Age (years)		
Mean age (years)	45.2±10.4	50.6±10.2
Median age (years)	46	50.5
Gender		
Male	35	2
Female	34	8
WHO grade		
Grade I	0	10
Grade II	30	0
Grade III	39	0
Pathological classification		
Astrocytoma	44	0
Oligodendroglioma	20	0
Mixed glioma	5	0
Benign meningioma	0	7
Schwannoma	0	3
Treatments history		
Surgery only	11	10
Surgery + radiotherapy	17	0
Surgery + chemotherapy	14	0
Surgery + chemoradiotherapy	27	0
IDH mutation status		
IDH mutation	26	0
IDH wild-type	28	0
Unknown	15	10
Seizure history		
Yes	35	4
No	34	6

LGG, Diffuse lower-grade gliomas; WHO, World Health Organization.

Supplementary Table S3. Detailed clinical data of patients with LGG and benign brain tumor

Subjects	*Gender*	*Age (years)*	*WHO grade*	*Pathological classification*	*Time of diagnosis*	*Surgery*	*Radiation*	*Chemotherapy*	*IDH_mutation*	*Seizure history*
0893032	MALE	41	WHO_II	Oligodendroglioma	Feb-13	YES	YES	NO	UNKNOWN	YES
0892976	FEMALE	63	WHO_III	Astrocytoma	Apr-13	YES	YES	YES	NO	NO
0897478	FEMALE	40	WHO_II	Astrocytoma	May-13	YES	YES	YES	NO	NO
0903584	MALE	46	WHO_III	Astrocytoma	Jun-13	YES	YES	NO	NO	YES
0971506	FEMALE	40	WHO_III	Oligodendroglioma	Jul-13	YES	YES	YES	YES	NO
1705404	MALE	39	WHO_II	Mixed glioma	Nov-13	YES	NO	NO	YES	YES
1057091	FEMALE	48	WHO_III	Astrocytoma	Feb-14	YES	YES	NO	NO	YES
1425317	FEMALE	46	WHO_II	Astrocytoma	Mar-14	YES	YES	NO	NO	NO
1166153	FEMALE	18	WHO_II	Astrocytoma	Mar-14	YES	YES	YES	NO	YES
0995481	MALE	54	WHO_II	Astrocytoma	Apr-14	YES	NO	NO	NO	NO
1179427	FEMALE	51	WHO_II	Astrocytoma	Jun-14	YES	YES	YES	UNKNOWN	NO
1022939	MALE	41	WHO_II	Oligodendroglioma	Jul-14	YES	NO	NO	YES	YES
1072091	MALE	58	WHO_III	Astrocytoma	Aug-14	YES	NO	YES	YES	NO
1141355	FEMALE	50	WHO_III	Astrocytoma	Aug-14	YES	YES	NO	UNKNOWN	YES
1052710	FEMALE	70	WHO_II	Astrocytoma	Sep-14	YES	NO	NO	UNKNOWN	NO
1054035	MALE	45	WHO_III	Astrocytoma	Sep-14	YES	NO	NO	YES	NO
1104236	MALE	48	WHO_II	Astrocytoma	Oct-14	YES	NO	YES	YES	YES
1216845	FEMALE	58	WHO_II	Astrocytoma	Oct-14	YES	YES	YES	YES	YES
1126176	MALE	55	WHO_II	Astrocytoma	Nov-14	YES	YES	YES	NO	NO
1116867	MALE	52	WHO_III	Astrocytoma	Nov-14	YES	NO	YES	UNKNOWN	NO
1128780	FEMALE	55	WHO_III	Astrocytoma	Nov-14	YES	YES	YES	YES	NO
1136777	MALE	52	WHO_II	Astrocytoma	Dec-14	YES	YES	NO	YES	NO
1089330	FEMALE	53	WHO_III	Astrocytoma	Dec-14	YES	YES	YES	NO	NO
1191328	MALE	33	WHO_III	Oligodendroglioma	Dec-14	YES	YES	YES	YES	YES
1525700	MALE	34	WHO_II	Oligodendroglioma	Feb-15	YES	YES	YES	UNKNOWN	NO
1507948	FEMALE	33	WHO_II	Oligodendroglioma	Apr-15	YES	YES	YES	YES	NO
1524925	MALE	32	WHO_III	Astrocytoma	Apr-15	YES	YES	YES	NO	YES
1246781	FEMALE	21	WHO_III	Mixed glioma	Apr-15	YES	NO	YES	NO	YES
1526406	FEMALE	63	WHO_III	Oligodendroglioma	May-15	YES	NO	YES	YES	YES
1514053	MALE	62	WHO_III	Astrocytoma	Jul-15	YES	NO	YES	NO	YES
1521011	MALE	47	WHO_III	Astrocytoma	Oct-15	YES	YES	YES	UNKNOWN	NO
1528077	MALE	29	WHO_II	Astrocytoma	Nov-15	YES	YES	YES	NO	NO
1529782	FEMALE	56	WHO_III	Astrocytoma	Nov-15	YES	YES	NO	YES	YES
1529856	FEMALE	45	WHO_III	Astrocytoma	Nov-15	YES	YES	YES	NO	YES
1627726	FEMALE	47	WHO_III	Oligodendroglioma	Jan-16	YES	YES	YES	UNKNOWN	YES
1602468	FEMALE	49	WHO_III	Oligodendroglioma	Mar-16	YES	YES	YES	NO	YES
1318011	MALE	47	WHO_II	Astrocytoma	Mar-16	YES	YES	YES	UNKNOWN	NO
1614542	FEMALE	46	WHO_III	Astrocytoma	Apr-16	YES	YES	YES	NO	NO
1618962	MALE	19	WHO_II	Mixed glioma	May-16	YES	NO	NO	YES	YES
1603963	FEMALE	22	WHO_III	Mixed glioma	May-16	YES	NO	YES	YES	YES
1338136	MALE	42	WHO_II	Oligodendroglioma	May-16	YES	NO	NO	YES	YES
1608207	FEMALE	45	WHO_III	Astrocytoma	Jul-16	YES	YES	NO	NO	YES
1620562	FEMALE	46	WHO_III	Astrocytoma	Oct-16	YES	YES	NO	UNKNOWN	NO
1631230	MALE	34	WHO_II	Astrocytoma	Dec-16	YES	YES	YES	YES	NO
1612120	FEMALE	43	WHO_III	Oligodendroglioma	Dec-16	YES	YES	NO	YES	NO
1718726	MALE	37	WHO_II	Oligodendroglioma	Feb-17	YES	NO	YES	YES	NO
1701459	FEMALE	61	WHO_III	Astrocytoma	Feb-17	YES	YES	NO	UNKNOWN	YES
1700259	MALE	49	WHO_III	Oligodendroglioma	Feb-17	YES	NO	YES	NO	YES
1701115	FEMALE	55	WHO_II	Astrocytoma	Mar-17	YES	YES	NO	YES	NO
1732393	MALE	46	WHO_II	Astrocytoma	May-17	YES	YES	NO	NO	NO
1700541	MALE	54	WHO_III	Mixed glioma	May-17	YES	YES	NO	YES	YES
1397071	FEMALE	30	WHO_I	Schwannoma	May-17	YES	NO	NO	UNKNOWN	NO
1710002	MALE	45	WHO_II	Astrocytoma	Jun-17	YES	NO	YES	YES	YES
1732065	MALE	39	WHO_II	Oligodendroglioma	Jun-17	YES	NO	YES	NO	NO
1421257	FEMALE	47	WHO_III	Astrocytoma	Jun-17	YES	YES	YES	YES	YES
1310257	MALE	46	WHO_III	Astrocytoma	Jul-17	YES	YES	YES	UNKNOWN	YES
1716600	MALE	41	WHO_II	Oligodendroglioma	Aug-17	YES	NO	YES	YES	NO
1436099	MALE	31	WHO_III	Astrocytoma	Aug-17	YES	YES	YES	NO	YES
1720885	MALE	46	WHO_III	Oligodendroglioma	Sep-17	YES	YES	YES	NO	YES
1736588	MALE	49	WHO_III	Astrocytoma	Oct-17	YES	YES	NO	NO	NO
1712584	MALE	52	WHO_III	Oligodendroglioma	Nov-17	YES	NO	NO	UNKNOWN	YES
1712826	MALE	54	WHO_III	Oligodendroglioma	Nov-17	YES	NO	YES	NO	NO
1738022	FEMALE	36	WHO_III	Oligodendroglioma	Dec-17	YES	YES	YES	NO	NO
1443128	MALE	42	WHO_I	Benign meningioma	Dec-17	YES	NO	NO	UNKNOWN	YES
1386295	FEMALE	61	WHO_I	Benign meningioma	Dec-17	YES	NO	NO	UNKNOWN	NO
1448208	FEMALE	67	WHO_I	Benign meningioma	Jan-18	YES	NO	NO	UNKNOWN	NO
1513827	FEMALE	55	WHO_I	Schwannoma	Jan-18	YES	NO	NO	UNKNOWN	NO
1814879	FEMALE	51	WHO_III	Astrocytoma	Feb-18	YES	NO	YES	UNKNOWN	YES
1441389	FEMALE	49	WHO_III	Astrocytoma	Feb-18	YES	NO	NO	NO	YES
1824897	FEMALE	48	WHO_III	Astrocytoma	Mar-18	YES	YES	YES	NO	NO
1461422	FEMALE	47	WHO_I	Benign meningioma	Mar-18	YES	NO	NO	UNKNOWN	NO
1469919	FEMALE	47	WHO_I	Benign meningioma	Apr-18	YES	NO	NO	UNKNOWN	YES
1475147	MALE	56	WHO_I	Schwannoma	Apr-18	YES	NO	NO	UNKNOWN	NO
1479762	FEMALE	49	WHO_I	Benign meningioma	May-18	YES	NO	NO	UNKNOWN	YES
1810160	MALE	35	WHO_II	Astrocytoma	Jun-18	YES	NO	NO	YES	NO
1812853	FEMALE	34	WHO_II	Astrocytoma	Jul-18	YES	YES	NO	NO	YES
1823881	FEMALE	46	WHO_II	Oligodendroglioma	Aug-18	YES	YES	NO	UNKNOWN	NO
1825660	FEMALE	48	WHO_II	Astrocytoma	Aug-18	YES	NO	NO	YES	YES
1531418	FEMALE	52	WHO_I	Benign meningioma	Oct-18	YES	NO	NO	UNKNOWN	YES

LGG, Diffuse lower-grade gliomas; WHO, World Health Organization.

Supplementary Figure S1.

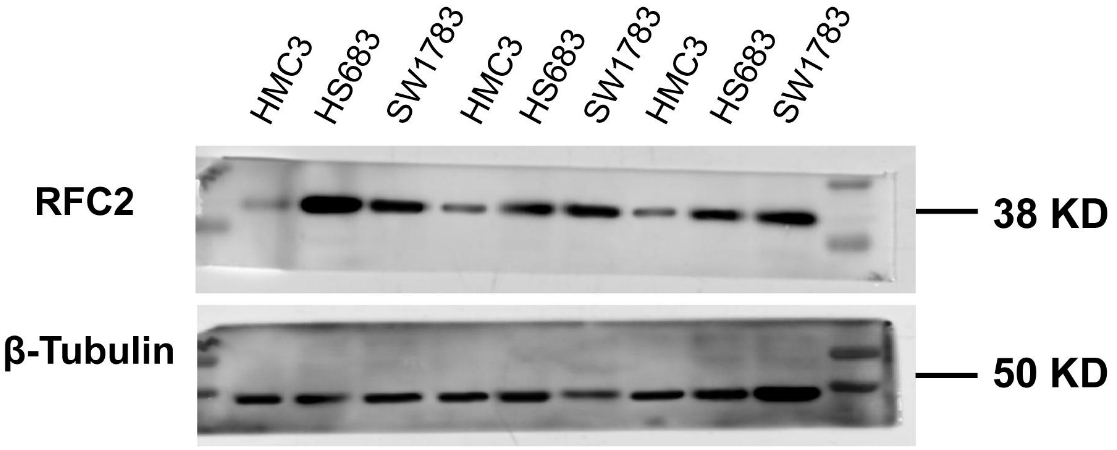

Supplementary Figure S1. The scans of membranes for Western blot images of RFC2 expression in HMC3, HS683, and SW1783 cells respectively corresponding to Fig. 10A.

Supplementary Figure S2.

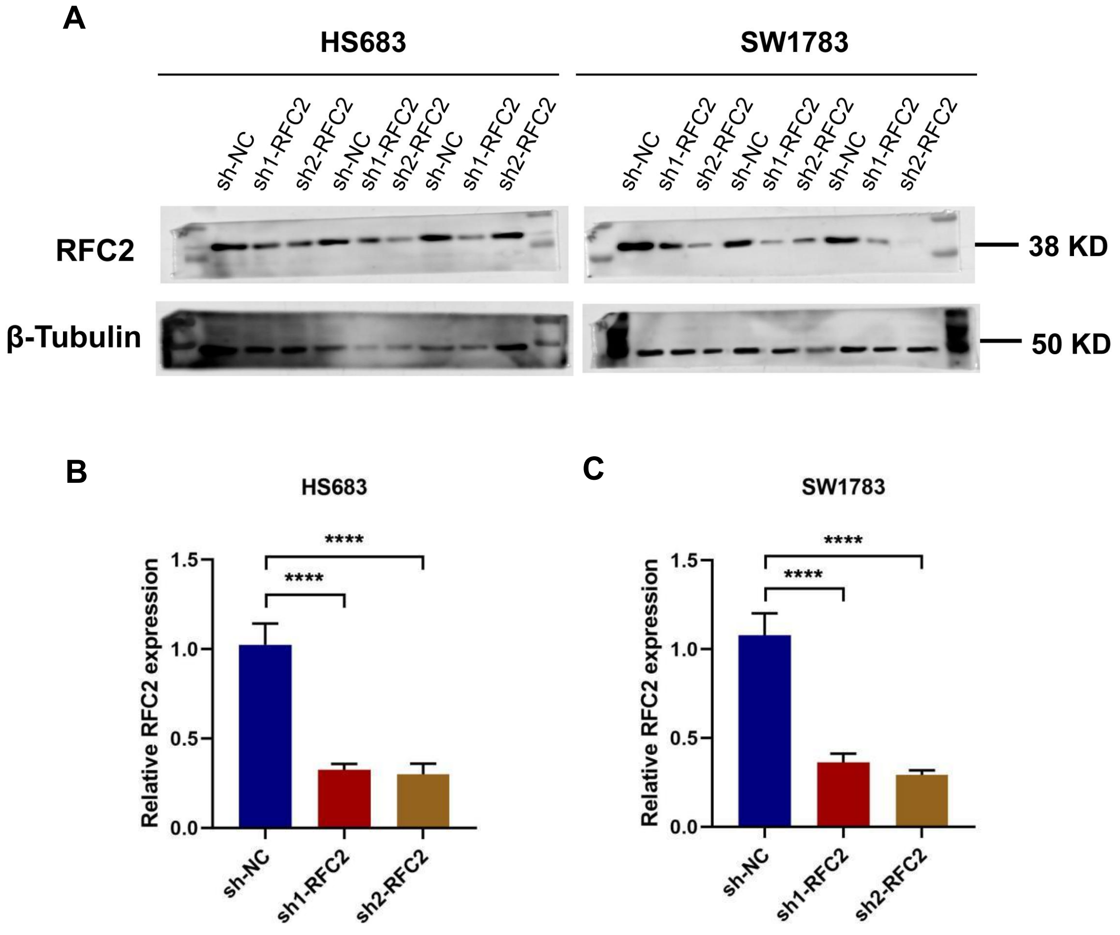

Supplementary Figure S2. Western blot and qRT-PCR were used to evaluate the expression levels of RFC2 in HS683 and SW1783 cells. (**A)** The scans of membranes for Western blot images of RFC2 expression in HS683 and SW1783 cells corresponding to Fig. 10C. (**B)** The relative expression of RFC2 in HS683 cells was evaluated by qRT-PCR. (**C)** The relative expression of RFC2 in SW1783 cells was evaluated by qRT-PCR. (*****P*<0.0001).
